# Integrative transcriptomic and metabolomic analyses of the drought response mechanisms of stem spines in desert plants from the Ebinur Lake

**DOI:** 10.3389/fpls.2026.1911473

**Published:** 2026-08-24

**Authors:** Meiyu Wang, Wenjing Li, Hengfang Wang, Guanghui Lv, Jianhao Li

**Affiliations:** 1 College of Ecology and Environment, Xinjiang University, Urumqi, Xinjiang, China; 2 Key Laboratory of Oasis Ecology of Education Ministry, Xinjiang University, Urumqi, China; 3 College of Geography and Remote Sensing Sciences, Xinjiang University, Urumqi, Xinjiang, China

**Keywords:** desert ecosystem, drought stress, metabolome, plant spines, transcriptome

## Abstract

Stem spines are key adaptive organs in desert plants, playing a critical role in resisting drought stress and maintaining plant survival. However, the molecular mechanisms governing their responses to drought remain insufficiently characterized. We investigated the stem spines of two representative halophytic shrubs from the Ebinur Lake Wetland desert region, *Alhagi sparsifolia* and *Nitraria tangutorum*, under a natural drought gradient: mild drought (Mi), 15.51% ± 0.61%; moderate drought (Mo), 8.87% ± 0.77%; severe drought (S), 3.34% ± 0.26%), and integrated transcriptomic and metabolomic analyses were performed to systematically reveal the molecular basis of stem spine responses to drought stress. The results showed that, compared with Mi, chlorophyll content in *N. tangutorum* stem spines decreased by 1.22- and 1.67-fold under Mo and S, respectively, whereas superoxide dismutase activity increased by 1.10-fold under Mo. Integrated transcriptome and metabolome analyses revealed that drought-responsive genes and metabolites under Mo and S were predominantly enriched in the isoflavonoid biosynthesis, arachidonic acid metabolism, steroid/brassinosteroid biosynthesis, and tryptophan metabolism pathways. In the tryptophan metabolism pathway, both metabolites and genes in *N. tangutorum* changed significantly, and tryptamine, auxin (IAA), and serotonin accumulated markedly under both Mo and S. In the isoflavonoid biosynthesis pathway, *N. tangutorum* exhibited increased genistein under Mo and elevated isoformononetin under S, whereas *A. sparsifolia* showed sustained accumulation of coumestrol and glyceollin II, with particularly strong increases in glyceollin II under both drought levels. This study revealed the molecular basis of drought responses in desert plant stem spines and offers insights into desert plant adaptation and ecosystem conservation strategies.

## 1 Introduction

Stem spines constitute a major adaptive functional structure formed by plants in response to arid habitats. In addition to providing mechanical defense (Zhang et al., 2022), they improve drought tolerance by limiting transpiration and shaping the surrounding microenvironment, and are thus recognized as an important ecological trait for adaptation to long-term drought conditions (Pei et al., 2024). As global climate change intensifies, extreme drought events are becoming more frequent and more severe (West et al., 2024). Drought has thus emerged as an important abiotic stress factor that constrains plant growth, distribution, and ecosystem function (Shaffique et al., 2024), making drought-adaptive structures such as stem spines increasingly important for plant environmental adaptation. Desert ecosystems, as integral components of the terrestrial biosphere, perform essential ecological functions in global carbon and water cycling and in sustaining biodiversity (Fa et al., 2018; Xu et al., 2023; Qiu et al., 2023). Against the backdrop of ongoing warming, vegetation in this region is experiencing greater survival stress, which in turn poses sustained challenges to ecosystem stability and functional maintenance (Ding et al., 2020; Xiao et al., 2023). Therefore, elucidating the drought-adaptation mechanisms of stem spines in desert plants is of great significance for understanding ecosystem functioning.

Previous studies have indicated that stem spine formation is co-regulated by developmental control and epigenetic mechanisms (Kumari and Jaiswal, 2025). Drought can stimulate the development of spinous structures in certain plants (Bezzalla et al., 2018). For instance, in Goji Berry, drought-induced alterations in leaf DNA methylation activate branch-spine development-related gene expression and thereby facilitate spine formation (Yang et al., 2022a), whereas in other xerophytic species, spinous structures have been shown to enhance stress tolerance by limiting transpiration and reinforcing defensive capacity (Pablo et al., 2019; Zhang et al., 2022). In addition to their structural functions, Drought generally induces osmotic imbalance and oxidative stress in plants. The accumulation of proline, soluble sugars, and other compatible solutes contributes to osmotic adjustment, thereby helping to maintain cellular water balance and membrane stability (Ozturk et al., 2021). Meanwhile, antioxidant enzymes play important roles in detoxifying drought-induced reactive oxygen species (ROS) and mitigating oxidative damage (Kumari et al., 2024). However, whether stem spines themselves participate in osmotic adjustment, ROS detoxification, or antioxidant defense remains largely unknown. It is also unclear whether these potential physiological functions vary with drought intensity or differ among desert shrub species.

In recent years, advances in multi-omics technologies have offered new perspectives for elucidating plant stress response mechanisms (Hong et al., 2020; Ye et al., 2026). Transcriptomics can reveal gene expression regulatory networks (Wang et al., 2024a), whereas metabolomics can reflect the ultimate physiological responses of plants to environmental changes (Ji et al., 2026). Abscisic acid (ABA) accumulation activates the *PYR/PYL–PP2C–SnRK2* signaling module and downstream *ABF/AREB* transcription factors, thereby regulating stomatal behavior and the expression of stress-responsive genes (Cutler et al., 2010; Zhu, 2016). In addition, transcription factor families such as *DREB, NAC, MYB, WRKY*, and *bZIP* participate in regulating drought-responsive genes involved in water transport, osmotic adjustment, antioxidant defense, and cellular protection (Prusty et al., 2024). Under drought stress, plants maintain energy supply and osmotic adjustment by activating glycolysis/gluconeogenesis and amino acid biosynthesis, while simultaneously accumulating antioxidant compounds such as flavonoids to scavenge reactive oxygen species, thereby alleviating oxidative damage (Hu et al., 2022; Yang and Lv, 2022). Although integrative transcriptomic and metabolomic approaches have been widely applied to the study of plant drought tolerance, most work has focused on conventional functional organs such as leaves and roots (Yin et al., 2024; Qi et al., 2025), with relatively little attention paid to specialized organs such as stem spines. Therefore, elucidating the transcriptional regulation and metabolic response characteristics of stem spines under drought stress is of great significance for deepening our understanding of drought adaptation mechanisms in desert plants.

Located in northwestern Xinjiang, the Ebinur Lake Wetland National Nature Reserve is a typical desert-wetland composite ecosystem and among the most environmentally sensitive areas in China’s arid zone (Lin and Xu, 2025). This region has long been subjected to multiple environmental stresses, including water deficit, soil salinization, intense radiation, and windblown sand activity, resulting in a distinctive vegetation type dominated by halophytic and xerophytic shrubs (Jiang et al., 2024; Xiang et al., 2024). Among these, *N. tangutorum* (D) and *A. sparsifolia* (L) are dominant desert shrubs in the region, both of which bear well-developed stem spines (Li et al., 2024a). However, molecular-level understanding of how the stem spines of these two desert species respond to drought. Stress remains limited.

Accordingly, this study used stem spines of *N. tangutorum* and *A. sparsifolia* from the Ebinur Lake Wetland National Nature Reserve as the research material, sampled along a natural drought gradient, and integrated physiological measurements with transcriptomic and metabolomic analyses to systematically examine the regulatory mechanisms governing spine responses to drought stress. This study focused on: (1) the response profiles of oxidative stress and antioxidant defense systems in stem spines under drought stress; (2) the key regulatory pathways associated with transcriptional and metabolic shifts in stem spines across different drought intensities; and (3) the roles of the isoflavonoid biosynthesis and tryptophan metabolism pathways in stem spine drought adaptation, together with their molecular regulatory mechanisms. This study aimed to uncover the molecular basis of stem spine involvement in drought adaptation in desert plants and to provide a theoretical foundation for understanding environmental adaptation strategies in desert species as well as for the conservation and restoration of arid-zone ecosystems.

## 2 Materials and methods

### 2.1 Study area description and sample collections

**Figure 1:**
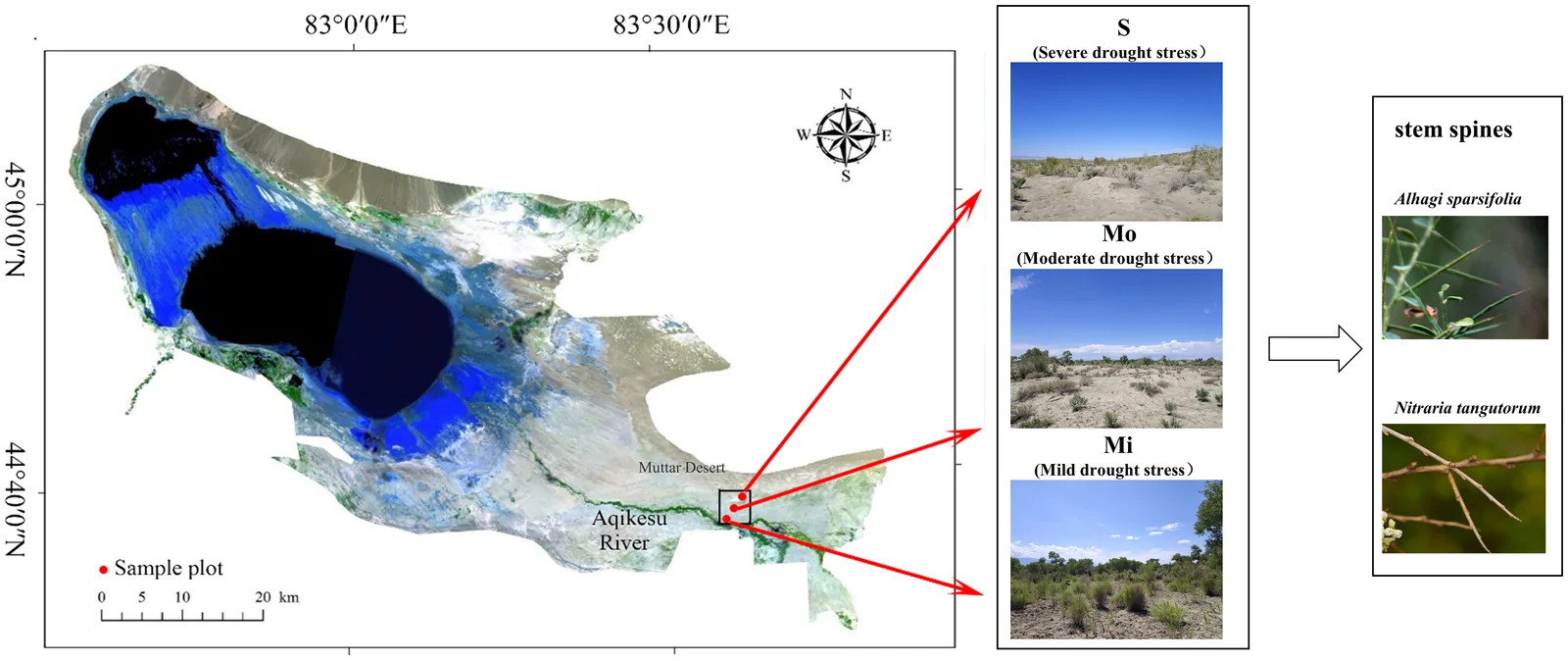
Overview map of the study area.

The Ebinur Lake Wetland National Nature Reserve, located in northwestern Xinjiang, China, is a typical desert-wetland transitional ecosystem (44°30′-45°09′ N, 82°36′-83°50′ E). A natural moisture-gradient transect of approximately 5 km was established between the north bank of the Aiqikusu River and the Mutetar Desert to represent the influence of groundwater-table depth on the water conditions of surface vegetation. Along the transect, three community survey plots measuring 30 m × 30 m were established at intervals of approximately 1.7 km in the direction of the moisture gradient (**Figure 1**). In the survey plots, groundwater depth decreased progressively with distance from the Aiqikusu River, thereby generating a continuous natural soil-moisture gradient (Yang et al., 2019). According to measurements from the 0–30 cm soil layer, the average soil moisture contents of the three plots were 15.51% ± 0.61%, 8.87% ± 0.77%, and 3.34% ± 0.26%, respectively. Based on the national standard for agricultural soil drought classification in China (GB/T 32136-2015), the above water contents corresponded to mild (Mi), moderate (Mo), and severe (S) drought stress, respectively (Yang et al., 2022b; Zhou et al., 2022; Li et al., 2022), and the three plots therefore represented a gradient of natural drought stress with increasing severity. The typical dominant desert shrubs *N. tangutorum* and *A. sparsifolia* were distributed across all three plots. Both species are widely distributed and highly adapted to arid environments. Their root dry matter content (RDMC) and specific root area (SRA) increased with increasing drought stress (Wang et al., 2025a). Taken together, the continuous moisture gradient and the coexistence pattern of typical dominant species render this region a stable and representative natural drought-gradient experimental system, offering an ideal field platform for investigating stem spine response mechanisms in desert plants under different drought intensities.

Stem spines were sampled after three consecutive sunny days. For each species in each plot, six individuals were selected (n=6), and stem spine samples were excised with sterile scissors and placed in sterile tubes or sterile plastic bags. After collection, samples were divided into four portions for metabolomic analysis, transcriptomic analysis, physiological assays, and measurement of osmotic adjustment substances, respectively. The samples designated for metabolomic, transcriptomic, and physiological analyses were immediately frozen in liquid nitrogen and stored at -80 °C in the laboratory; an additional portion was first blanched at 105 °C for 10 min and then dried at 65 °C to a constant mass.

### 2.2 Determination of biochemical parameters

To systematically evaluate the effects of drought stress on osmotic adjustment substances, oxidative-stress responses, and photosynthetic function in stem spines, the relevant physicochemical indices were determined using either dry or fresh samples. Soluble sugars and starch were quantified from dried material by the anthrone assay (Zhao et al., 2024), while the remaining parameters were determined from fresh tissue. Soluble protein was measured using the Coomassie brilliant blue assay, proline using the ninhydrin colorimetric method, SOD activity using the nitrogen blue tetrazolium (NBT) assay, and Malondialdehyde (MDA) content using the thiobarbituric acid (TBA) assay (Kumari et al., 2024). In terms of photosynthetic pigments, chlorophyll was extracted with 90% (v/v) ethanol; absorbance was then measured at 665, 649, and 470 nm to calculate chlorophyll a (chla), chlorophyll b (chlb), and carotenoid contents.

### 2.3 Transcriptome analysis

Six biological repeats were set for each drought gradient. The samples were sent to Shanghai Meggie Biomedical Technology Co., Ltd. for transcriptome sequencing (RNA-seq) and metabonomics analysis (LC-MS) respectively. Total RNA was extracted from tissue samples, the concentration and purity of RNA were detected by NanoDrop 2000, and the integrity of RNA was preliminarily detected by agarose gel electrophoresis. High-throughput sequencing was performed on the Illumina Nova Seq X Plus platform to obtain the original reads. Then, the original sequencing data were subjected to quality control, linker sequence removal and low-quality sequence filtering using fastp to obtain clean reads. Clean reads was assembled from scratch by Trinity software, and transcripts and unigenes were obtained, which were used for subsequent gene expression quantification and functional analysis (Grabherr et al., 2011). DESeq2 was used to screen differentially expressed genes between groups, and ∣log_2_(FC)∣>1 and corrected P<no><0.05</no> was used as the screening criteria for differentially expressed genes (Rao et al., 2022). Furthermore, GO function annotation and KEGG pathway enrichment analysis of differentially expressed genes were carried out to clarify the regulatory effect of drought stress on transcription level and its related biological processes.

### 2.4 Metabolomic analysis

An accurately weighed 50 mg aliquot was extracted with 400 μL of 80% (v/v) methanol, homogenized in a cryogenic tissue grinder at 50 Hz for 6 min, and then sonicated at low temperature for 30 min to maximize metabolite recovery. The extract was incubated at -20 °C for 30 min and then centrifuged at 4 °C at 13,000 × g for 15 min, after which the supernatant was used for LC-MS detection. Differential accumulation metabolites (DAMs) were screened by constructing an OPLS-DA discriminant model based on the metabolomic profiles, which maximized the separation among treatment groups and captured the key discriminatory metabolic signals (Liu et al., 2025). DAMs were defined as those with VIP > 1 and FC > 1.2 or FC < 5/6. Subsequently, the KEGG database was employed for functional annotation and pathway enrichment analysis of the DAMs. In addition, Venn diagrams were constructed to identify common and unique DAMs between the two pairwise comparison groups, thereby informing downstream mechanistic analysis.

### 2.5 Data analysis

Principal component analysis (PCA) and Spearman correlation analysis were used to assess the reproducibility of the transcriptomic data and the consistency of QC samples, thereby verifying the inter-sample correlation structure and ensuring a robust basis for downstream analyses. For multi-omics integration, DEGs and DAMs were subjected to joint pathway analysis, and significantly enriched pathways were screened using a hypergeometric test to identify the key biological processes underlying the treatment response, especially those involved in drought-adaptation metabolism and regulation (Liu et al., 2025). All statistical analyses and visualizations were carried out in R 4.5.0, using ggplot2 and car for data processing and figure generation. Data are expressed as mean ± SD, and group differences were assessed by one-way ANOVA, with *P < 0.05* considered statistically significant.

## 3 Results

### 3.1 Physiological response of stem thorns under different drought stress

**Figure 2:**
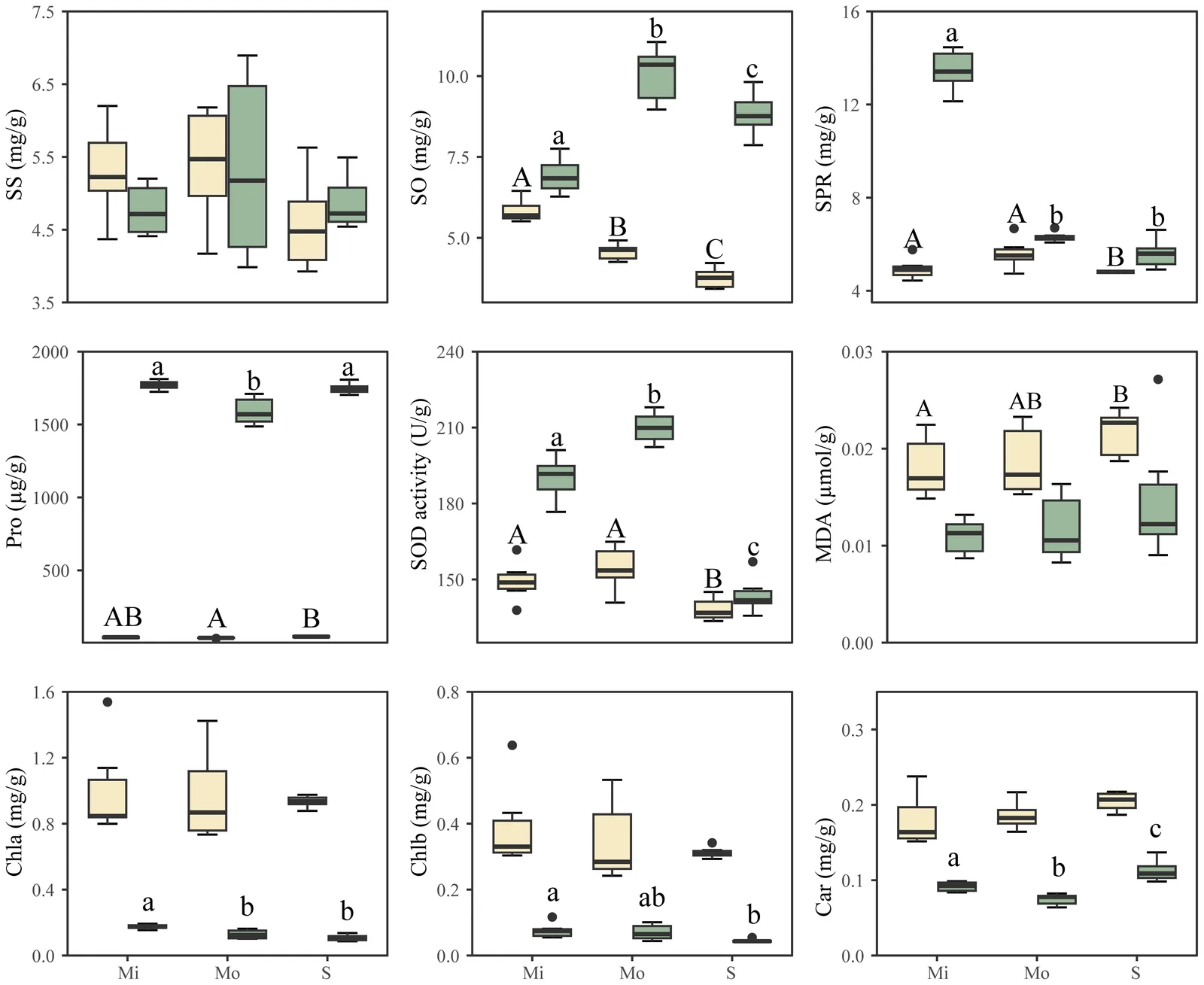
Physiochemical differences in stem spines of *A. sparsifolia* and *N. tangutorum* under drought stress. Mi, Mo, and S correspond to mild, moderate, and severe drought stress. Soluble sugar content (SS), starch oxide (SO),Soluble protein (SPR), proline content (Pro), malondialdehyde content (MDA), chlorophyll a content (Chla), chlorophyll b content (Chlb). and carotenoid content (Car). uppercase letters (A, B, C) denote the significant differences among drought gradients for *A. sparsifolia*, while lowercase letters (a, b, c) denote those for *N. tangutorum*.

Stem spine physiological traits differed significantly between the two species across drought treatments (**Figure 2**) (*P < 0.05*). In *N. tangutorum*, starch content was 10.06 mg/g under Mo and 8.82 mg/g under S, corresponding to 1.45- and 1.28-fold increases relative to Mi, respectively; SOD activity was 154.38 U/g under Mo, 1.10-fold higher than under Mi; chla and chlb contents declined as drought intensity increased, reaching 0.13 mg/g and 0.11 mg/g under Mo and S, respectively, which were 1.36- and 1.63-fold lower than under Mi; meanwhile, carotenoid content under S was 0.11 mg/g, representing a 1.23-fold increase. In *A. sparsifolia*, MDA content rose progressively with drought intensity, increasing by 1.29-fold under S, and proline content increased by 1.11-fold under S. Soluble sugar content showed no significant differences across drought treatments, while soluble protein content declined as drought stress intensified. The relative water content of the stem spines decreased significantly with increasing drought stress (**Supplementary Figure 1**).

### 3.2 Overview of RNA-seq results

**Figure 3:**
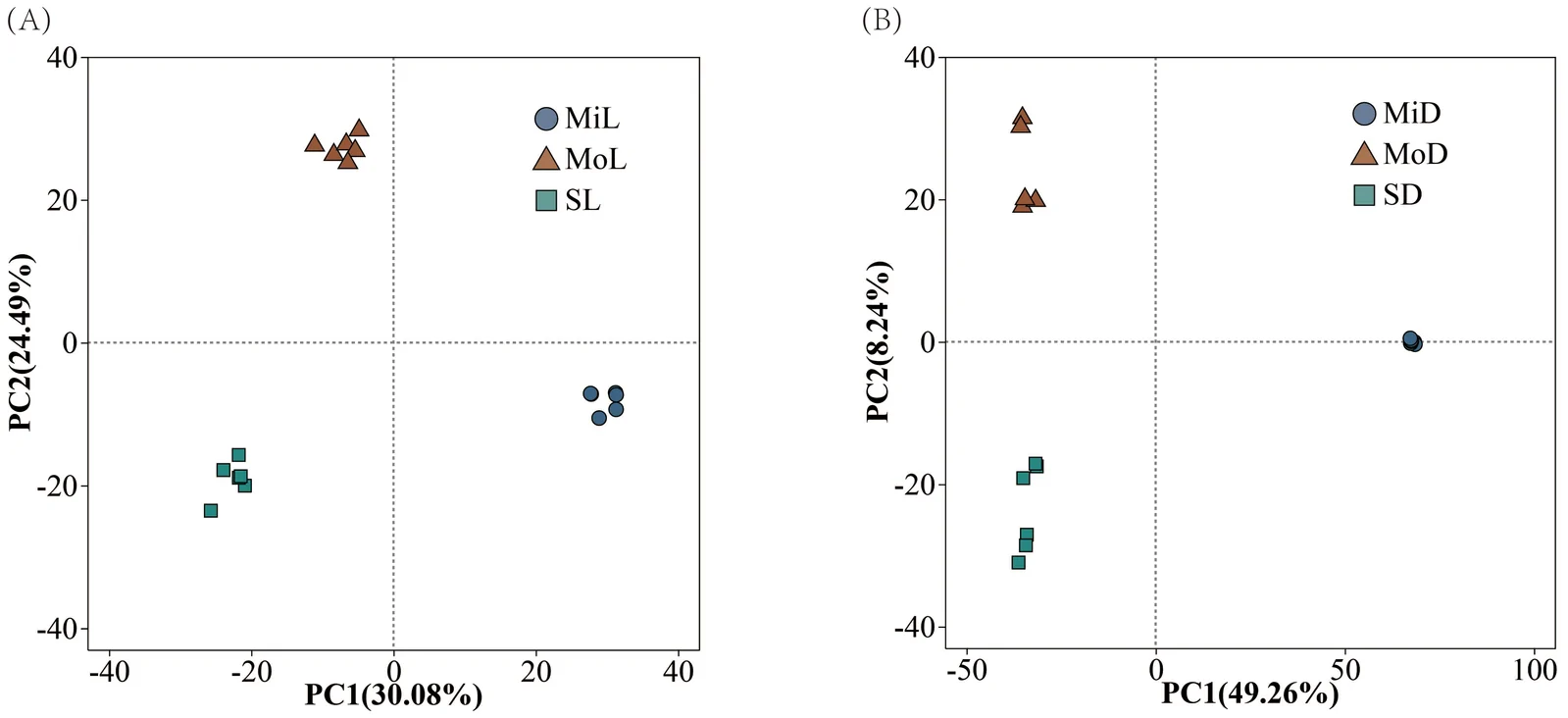
PCA of RNA-seq data. **(A)** *A. sparsifolia*; **(B)** *N. tangutorum*.

To elucidate the molecular basis of drought tolerance in stem spines, transcriptome sequencing was conducted on *A. sparsifolia* and *N. tangutorum* stem spines collected across different drought gradients. Using the Illumina NovaSeq platform and a paired-end sequencing strategy, 36 cDNA libraries were constructed and sequenced. Following rigorous quality control, 217.33 Gb of high-quality valid data were generated, with each sample yielding more than 5.42 Gb of clean reads and a Q30 score of 96.24%. PCA of the RNA-seq dataset indicated that PC1 accounted for 30.08% and 49.26% of the variance in stem spines of *A. sparsifolia* and *N. tangutorum* across drought gradients, respectively (**Figure 3**). In addition, the Pearson correlation coefficients (PCCs) among samples from different drought gradients ranged from 0.256 to 0.899 and from 0.235 to 0.909, respectively (**Supplementary Figure 2**).

### 3.3 Identification of DEGs, GO annotation, and GO enrichment

**Figure 4:**
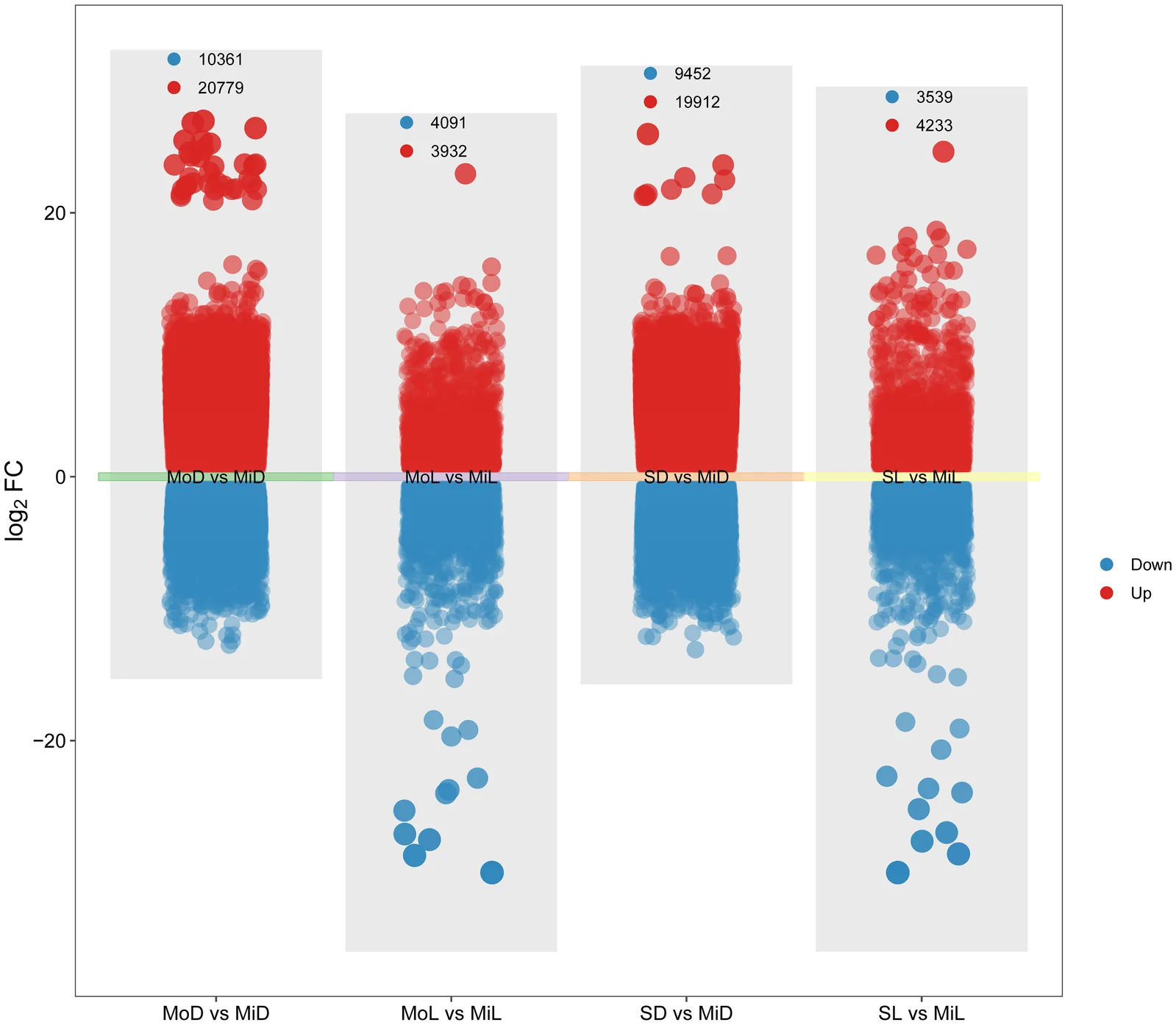
Volcano plot of DEGs.

Volcano plot analysis showed pronounced differences in the distribution of DEGs in stem spines across drought gradients (**Figure 4**). In *A. sparsifolia*, the MoL vs. MiL comparison identified 8023 DEGs (3932 upregulated and 4091 downregulated), whereas the SL vs. MiL comparison identified 7772 DEGs (4233 upregulated and 3539 downregulated), suggesting an increase in upregulated genes under S. In contrast, *N. tangutorum* stem spines exhibited 31140 DEGs in the MoD vs. MiD comparison (20779 upregulated and 10361 downregulated) and 29364 DEGs in the SD vs. MiD comparison (19912 upregulated and 9452 downregulated), with fewer upregulated and downregulated DEGs under S. Venn diagram analysis indicated that *A. sparsifolia* contained 11954 shared DEGs, of which 3841 were common DEGs, whereas *N. tangutorum* contained 37662 shared DEGs, including 22842 common DEGs (**Supplementary Figure 3**). GO annotation revealed that DEGs in *A. sparsifolia* under drought stress were primarily classified into cellular component, biological process, and molecular function categories (**Supplementary Figure 4**), whereas DEGs in *N. tangutorum* were mainly assigned to biological process and molecular function categories (**Supplementary Figure 5**). GO enrichment analysis further indicated significant enrichment of DEGs associated with response to stimulus in *N. tangutorum*, while transport-related DEGs were more strongly enriched in *A. sparsifolia*.

### 3.4 KEGG pathway analysis of DEGs

**Figure 5:**
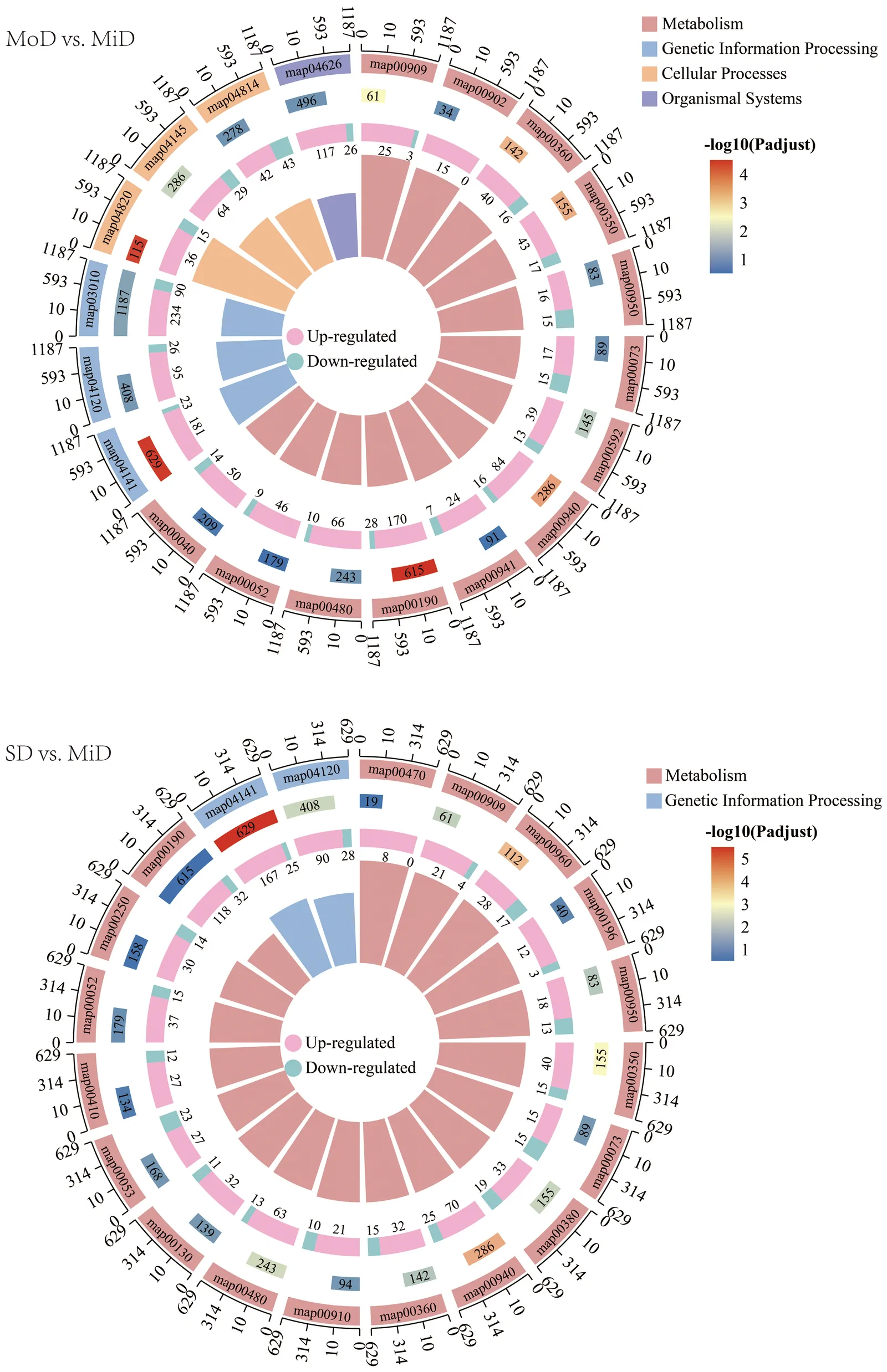
KEGG enrichment of DEGs in *N. tangutorum* stem spines. From the innermost circle outward, the circles represent the functional classification of the enriched genes, the number of genes enriched in each category, the proportions of up- and down-regulated genes, and the enrichment ratio (rich factor) for each term.

KEGG enrichment analysis identified 1672 and 1469 DEGs in *A. sparsifolia* for the MoL vs. MiL and SL vs. MiL comparisons, respectively, with significant enrichment in 137 and 136 KEGG pathways (**Supplementary Figure 6**); in *N. tangutorum*, 6104 and 5504 DEGs were identified in the MoD vs. MiD and SD vs. MiD comparisons, respectively, and were significantly enriched in 140 and 142 KEGG pathways (**Figure 5**). Further analysis indicated that DEGs in *A. sparsifolia* were predominantly enriched in plant hormone signal transduction, starch and sucrose metabolism, and plant-pathogen interaction pathways, while DEGs in *N. tangutorum* were mainly enriched in oxidative phosphorylation.

### 3.5 Metabolomic profiling

**Figure 6:**
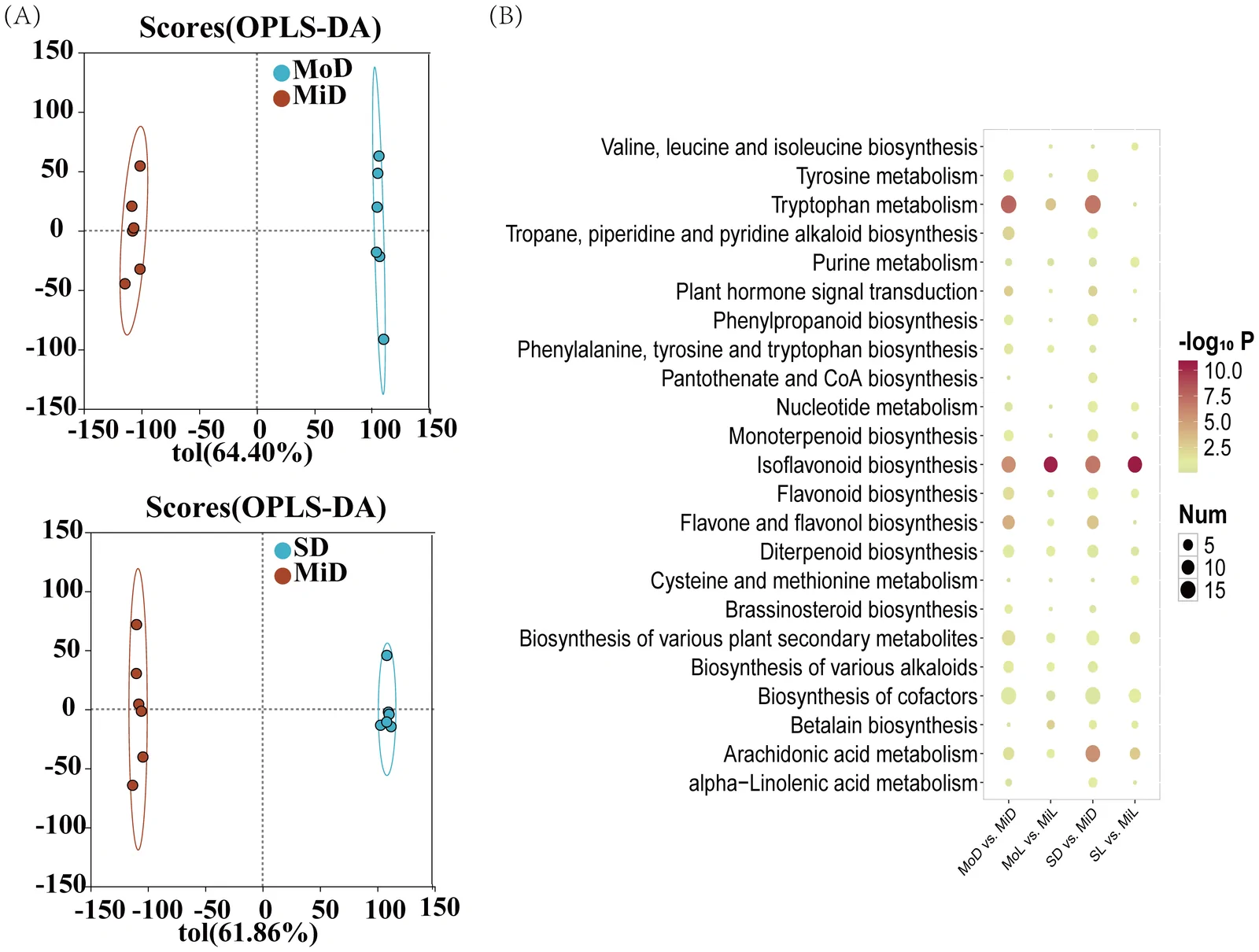
Identification and KEGG analysis of DAMs in stem spines. **(A)** OPLS-DA score plots for MoD vs. MiD and SD vs. MiD. **(B)** KEGG enrichment of DAMs. Color and bubble size represent the *P* value and the number of enriched genes in each pathway, respectively.

PCA showed that the first principal component (PC1) explained 30.20% and 55.00% of the total variance in the stem spines of *A. sparsifolia* and *N. tangutorum*, respectively (**Supplementary Figure 7**).The OPLS-DA score plots showed clear separation among samples on the first two axes, and the combined explanatory power of the first two axes for MoL vs. MiL, SL vs. MiL, MoD vs. MiD, and SD vs. MiD was 61.30%, 61.60%, 64.40%, and 61.86%, respectively (**Figure 6A**; **Supplementary Figure 8A**), indicating that different drought stress levels substantially altered the composition of metabolites associated with drought adaptation and that the metabolic profiles differed markedly among groups. In *A. sparsifolia*, the MoL vs. MiL comparison identified 536 DAMs (319 upregulated and 217 downregulated), whereas the SL vs. MiL comparison identified 618 DAMs (357 upregulated and 261 downregulated) (**Supplementary Figure 9**). The two comparisons shared 266 common DAMs (**Supplementary Figure 8B**). KEGG enrichment analysis indicated that these DAMs were primarily enriched in isoflavonoid biosynthesis, plant secondary metabolite biosynthesis, amino acid metabolism, fatty acid metabolism, and signal transduction pathways (**Figure 6B**). Because these pathways are broadly implicated in plant stress regulation, the results suggest that *A. sparsifolia* primarily responds to drought by remodeling secondary metabolism, lipid metabolism, and associated signaling networks to maintain physiological homeostasis and improve environmental adaptability. In *N. tangutorum*, the MoD vs. MiD comparison identified 1491 DAMs (998 upregulated and 493 downregulated), and the SD vs. MiD comparison identified 1555 DAMs (1153 upregulated and 402 downregulated) (**Supplementary Figure 9**), with 1253 DAMs shared between the two comparisons (**Supplementary Figure 8B**). KEGG enrichment analysis further indicated that these DAMs were mainly enriched in tryptophan metabolism, isoflavonoid biosynthesis, and flavone and flavonol biosynthesis pathways (**Figure 6B**). Across drought intensities, *N. tangutorum* mainly improved antioxidant defense and stress tolerance by modulating aromatic amino acid metabolism and flavonoid-related secondary metabolism.

### 3.6 Integrated transcriptomic and metabolomic analysis

**Figure 7:**
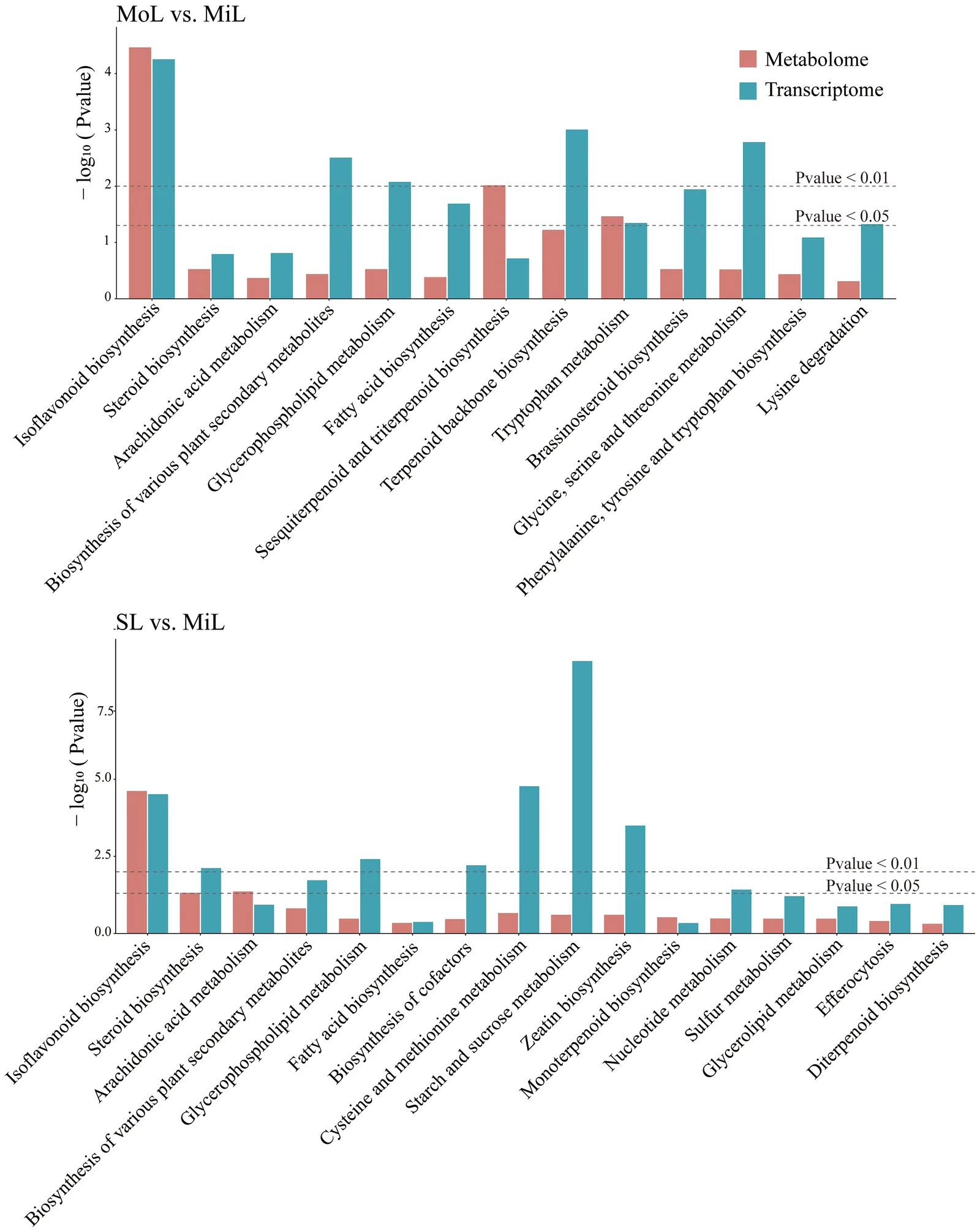
KEGG enrichment analysis of the top 20 significantly enriched pathways for DEGs and DAMs in *A. sparsifolia* stem spines.

To more directly visualize expression-pattern differences among significantly altered genes and metabolites, hierarchical clustering analysis was conducted based on expression levels (**Supplementary Figure 10**). The results indicated a positive correlation in the regulation of these genes and metabolites, implying that metabolite changes may be positively driven by gene regulation. Combined KEGG enrichment analysis of DEGs and DAMs indicated that MoL vs. MiL and SD vs. MiD were mainly enriched in isoflavonoid biosynthesis, steroid biosynthesis, arachidonic acid metabolism, glycerophospholipid metabolism, and biosynthesis of various plant secondary metabolites (**Figure 7**), pathways that are largely linked to plant adaptive responses to drought stress. Meanwhile, the KEGG enrichment results for MoD vs. MiD and SD vs. MiD revealed significant enrichment of tryptophan metabolism, isoflavonoid biosynthesis, plant hormone signal transduction, and arachidonic acid metabolism (**Supplementary Figure 11**). Notably, within tryptophan metabolism, tryptophan is a precursor of IAA (auxin), while aromatic pathways can produce flavonoids and phenylpropanoids, influencing growth regulation and antioxidant defense. Under drought stress, isoflavonoid biosynthesis contributes to antioxidant defense, ROS scavenging, membrane stabilization, and protection against UV and photoinhibition.

### 3.7 Changes in key genes and metabolites in isoflavonoid biosynthesis and tryptophan metabolism under drought stress

**Figure 8:**
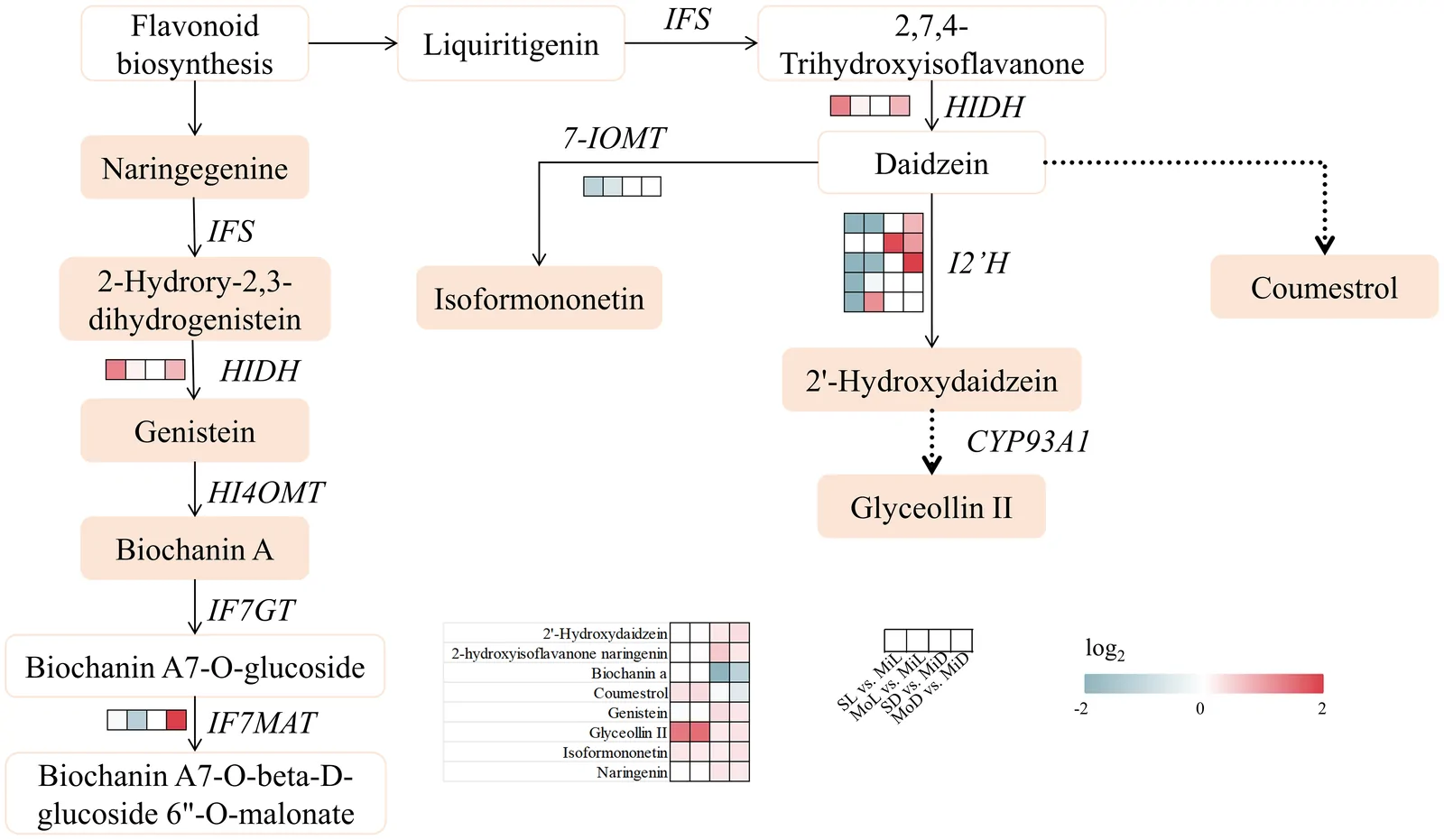
Integrated visualization of gene expression and metabolite accumulation in the isoflavonoid biosynthesis pathway of *A. sparsifolia* and *N. tangutorum* stem spines. The differential expression levels of the annotated genes are shown as heatmaps at their corresponding positions, with colors ranging from blue (low expression) to red (high expression). Metabolites are represented by squares of different colors. White squares indicate metabolites without annotations, whereas orange squares indicate annotated metabolites.

In the isoflavonoid biosynthesis pathway, *A. sparsifolia* stem spines showed pronounced metabolic activation under drought stress. Relative to MiL, coumestrol increased by 1.25- and 1.30-fold under MoL and SL, respectively, while glyceollin II increased by 2.67- and 2.85-fold, respectively, suggesting that drought promoted the accumulation of defense-associated isoflavonoid metabolites. In *N. tangutorum*, the response pattern of isoflavonoid metabolites was treatment-specific: under MoD vs. MiD, 2-hydroxyisoflavanone naringenin, genistein, and naringenin increased by 1.47-, 1.30-, and 1.21-fold, respectively; under SD vs. MiD, 2′-hydroxydaidzein, glyceollin II, and isoformononetin increased by 1.27-, 1.23-, and 1.23-fold, respectively. At the same time, pathway-related genes in *N. tangutorum* overall exhibited an upregulation trend (**Figure 8**), indicating that drought stress may enhance the accumulation of defensive secondary metabolites by activating the isoflavonoid biosynthetic network.

**Figure 9:**
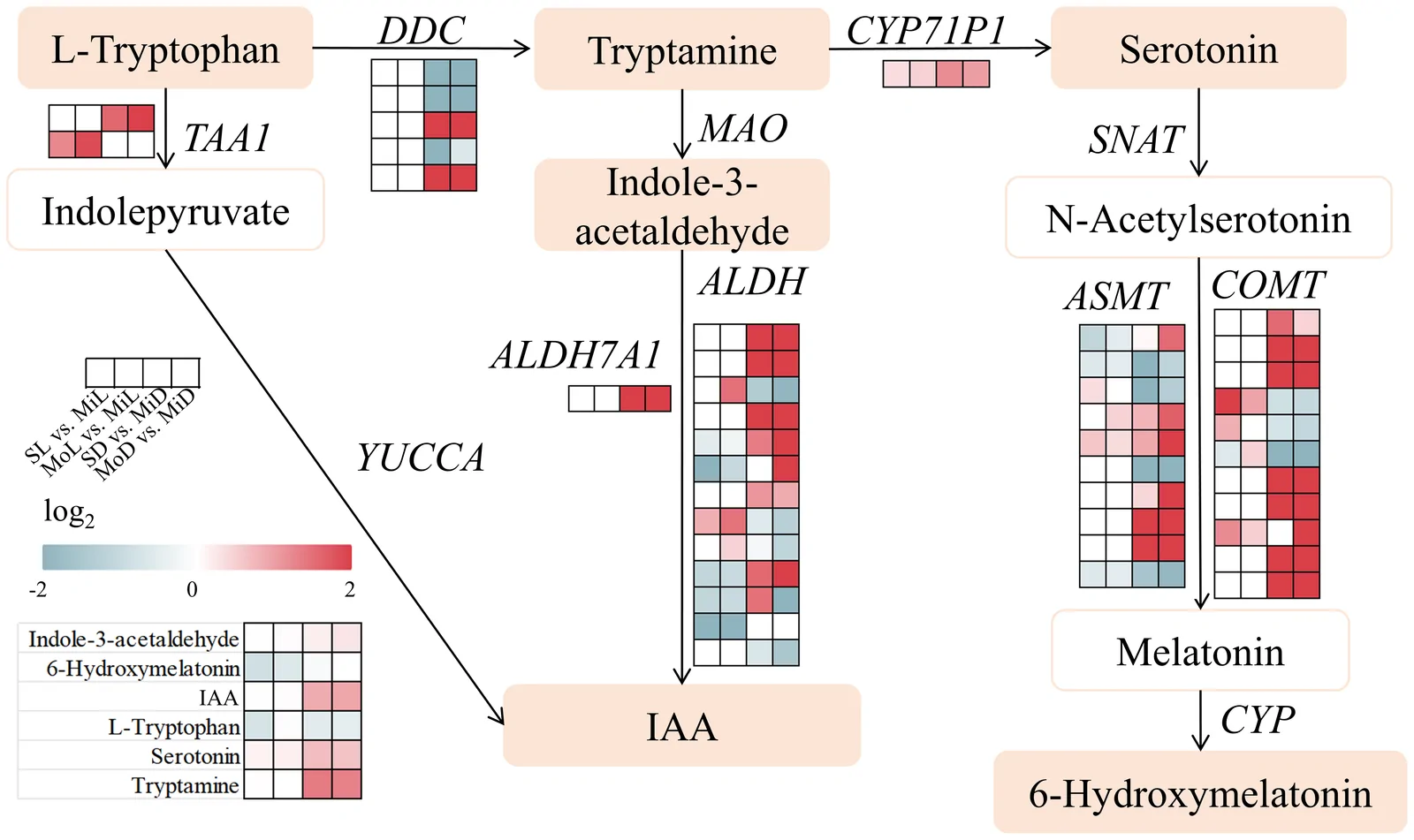
Integrated visualization of gene expression and metabolite accumulation in the tryptophan metabolism pathway of *A. sparsifolia* and *N. tangutorum* stem spines. The differential expression levels of the annotated genes are shown as heatmaps at their corresponding positions, with colors ranging from blue (low expression) to red (high expression). Metabolites are represented by squares of different colors. White squares indicate metabolites without annotations, whereas orange squares indicate annotated metabolites.

In the tryptophan metabolism pathway, *N. tangutorum* likewise showed marked metabolic reprogramming in response to drought stress. Relative to MiD, tryptamine increased by 2.61- and 2.47-fold in MoD and SD, respectively, IAA increased by 1.84- and 1.92-fold, respectively, and serotonin increased by 1.62- and 1.56-fold, respectively. At the same time, the tryptophan decarboxylase gene (*DDC*) was significantly upregulated in MoD and SD relative to MiD, and the aldehyde dehydrogenase gene (*ALDH*) and aldehyde dehydrogenase family 7 member A1 (*ALDH7A1*) also showed upward trends. Moreover, acetylserotonin O-methyltransferase (*ASMT*) and caffeic acid O-methyltransferase (*COMT*), both associated with melatonin metabolism, were also upregulated in MoD and SD (**Figure 9**). These findings indicate that under drought stress, *N. tangutorum* activates tryptophan-derived metabolism to accumulate key stress-adaptive metabolites.

## 4 Discussion

### 4.1 Physiological responses of stem spines under drought stress

As drought stress intensified, the decline in stem-spine relative water content indicated that the plants were experiencing water deficit, which may promote ROS accumulation and oxidative damage (She et al., 2024). Dynamic adjustment of the antioxidant enzyme system is generally a key physiological foundation of plant drought acclimation (Chen et al., 2016). Among the antioxidant defenses, SOD acts as a frontline component of the defense system by catalyzing the dismutation of superoxide anion radicals (O^2–^) into hydrogen peroxide, thereby mitigating damage arising from ROS overaccumulation (Khan et al., 2021). Previous studies have shown that drought can significantly increase SOD activity in plants such as *Triticum aestivum* and *Dioscorea esculenta* (Mishra and Panda, 2017; Melandri et al., 2020), indicating that enhanced SOD activity is usually closely associated with improved capacity to alleviate oxidative stress (Bashir et al., 2025). In *N. tangutorum*, SOD activity was significantly elevated under Mo, suggesting that the species may delay oxidative damage by activating antioxidant enzyme defenses to eliminate excess O^2–^; this response may represent a key physiological adjustment for coping with Mo.

Consistent with this, chloroplasts and their pigment systems exhibit high sensitivity to drought stress (Karami et al., 2025). Generally, drought stress suppresses chlorophyll synthesis while accelerating its degradation, which in turn inhibits photosynthesis and constrains plant growth (Oukarroum et al., 2007). Yan et al. (2024) further suggested that Mi may first trigger protective responses that help maintain relatively stable chla and chlb contents; as stress intensifies, however, chloroplast metabolism and structure may suffer sustained damage, ultimately resulting in decreased chlorophyll levels. In this study, chlorophyll content in *N. tangutorum* stem spines decreased progressively as drought intensified, suggesting that its photosynthetic apparatus is highly sensitive to water deficit and was substantially suppressed under Mo to S (Jiang et al., 2024).

### 4.2 Multi-omics integration of key metabolic pathways underlying drought tolerance

Joint analysis of DEGs and DAMs revealed substantial metabolic reprogramming in stem spines under different drought stresses, with several stress-adaptation-related pathways enriched in different comparison groups, including isoflavonoid biosynthesis, steroid/brassinosteroid biosynthesis, tryptophan metabolism, fatty acid biosynthesis/biosynthesis of unsaturated fatty acids, and biosynthesis of various plant secondary metabolites.

The enrichment of isoflavonoid biosynthesis and biosynthesis of various plant secondary metabolites suggests that stem spines may improve stress resistance by promoting secondary metabolite accumulation (Quan and Liu, 2024; Lv et al., 2025). Isoflavonoids are key antioxidant secondary metabolites capable of scavenging ROS, protecting membrane systems and photosynthetic complexes, and contributing to photoprotection and UV defense (Devi et al., 2020); thus, activation of this pathway can be viewed as a typical chemical antioxidant response under drought, which is often accompanied by photoinhibition and oxidative stress (Huang et al., 2023). These metabolic shifts indicate that stem spines may mitigate drought-induced oxidative injury and sustain cellular homeostasis by enhancing the biosynthesis of antioxidant secondary metabolites. At the same time, enrichment of fatty acid biosynthesis and biosynthesis of unsaturated fatty acids suggests that stem spines may preserve membrane stability and fluidity by modulating membrane lipid composition (Karaca-Bulut et al., 2025). Drought-triggered lipid remodeling, particularly an increase in unsaturated fatty acid proportion, helps alleviate membrane damage under dehydration and preserves membrane integrity and function (Liu et al., 2024). Thus, alterations in lipid metabolism are linked not only to membrane protection but may also supply a metabolic foundation for stress signaling.

Moreover, enrichment of steroid/brassinosteroid biosynthesis suggests that BR-associated metabolism may contribute to the regulation of stem spine adaptation to drought (Li et al., 2024b). BRs regulate not only cell elongation, photosynthetic performance, and antioxidant systems, but also interact with hormones such as ABA and IAA to coordinate resource allocation and physiological balance between growth and stress tolerance (Li et al., 2024b; Mahmood et al., 2024), indicating that stem spines may remodel sterol/BR metabolism to mediate the trade-off between growth suppression and defense enhancement under drought. Tryptophan is both essential for normal plant growth and development and a precursor for numerous key metabolites, contributing to the biosynthesis of hormones such as IAA, ABA, and SA, as well as secondary metabolites including chlorophyll, flavonoids, and alkaloids; thus, it occupies a pivotal position in plant adaptation to abiotic stress (Wang et al., 2025b).

### 4.3 Isoflavonoid biosynthesis and tryptophan metabolism involved in stem spine drought tolerance

As a flavonoid subclass enriched in legumes, isoflavonoids are pivotal to improving plant adaptation to abiotic stresses, particularly drought stress (Huang et al., 2023; Zhou et al., 2026). Among them, genistein is considered a key isoflavonoid in drought response, and its antioxidant activity allows it to mitigate drought injury by modulating reactive oxygen species homeostasis, strengthening antioxidant defenses, and sustaining photosynthetic stability (Yang et al., 2021; Tian et al., 2014; Zhou et al., 2024). In this study, genistein was significantly accumulated in the MoD vs. MiD comparison, while isoformononetin and HDIH were significantly upregulated in the SD vs. MiD comparison, suggesting that stem spines respond to drought of varying intensity not through a single mode, but through branched and stage-dependent reprogramming of the isoflavonoid pathway. Specifically, the increase in genistein under moderate drought may reflect the preferential activation of isoflavonoid defense metabolism; under severe drought, the simultaneous upregulation of isoformononetin and HDIH suggests further enhancement of the corresponding biosynthetic branch and its transcriptional regulation, thereby supporting the maintenance of redox balance and improving drought tolerance under stronger stress (Gümüş et al., 2026). Notably, in *A. sparsifolia*, coumestrol and glyceollin II also varied significantly among drought comparison groups, indicating that isoflavonoid metabolism in desert legumes may display both conservation and species-specific responsiveness. Previous studies have demonstrated that coumestrol enhances drought tolerance by activating ABA signaling, inducing stomatal closure, and reducing water loss (Ye et al., 2025), whereas glyceollin II may not directly regulate drought responses but can improve resistance to pathogen invasion under drought conditions (Correa-Molinari et al., 2021). Thus, the enhancement of isoflavonoid metabolism may represent not only a chemical defense response of stem spines to drought but also a contribution to antioxidant defense, stomatal regulation, and defense homeostasis through functional diversification of distinct derivatives.

Tryptophan metabolism is important in plant responses to abiotic stress, as its metabolites enhance stress adaptation by modulating growth, signaling, and antioxidant defense (Wang et al., 2025b). IAA, serotonin, and melatonin are key molecules in this pathway and are tightly linked in plant stress responses. IAA enhances antioxidant mechanisms and limits excessive accumulation of ROS and RNS in cells (Singh et al., 2021; Mittler et al., 2022); serotonin induces activities of antioxidant enzymes such as catalase, SOD, peroxidase, and glutathione reductase to mitigate oxidative injury (Liu et al., 2021; Akcay and Okudan, 2023); and melatonin directly scavenges ROS/RNS while reinforcing both enzymatic and non-enzymatic antioxidant systems (Wang et al., 2024b; Khan et al., 2025; Wu et al., 2025). At the same time, as a precursor of melatonin, serotonin may act together with melatonin in plant stress regulation. The significant enrichment of the tryptophan metabolism pathway in this study suggests that stem spines may modulate stress responses by reprogramming tryptophan-derived metabolism under drought. Previous studies have shown that melatonin can promote IAA biosynthesis by upregulating TAA1 under drought (Wei et al., 2022), while TDC induction can enhance serotonin accumulation and influence IAA transport and signaling (Mukherjee et al., 2014; Wan et al., 2018; Negri et al., 2021); however, these effects have been reported mainly under salt and oxidative stress, and their regulatory mode under drought remains unresolved. Thus, the enhancement of tryptophan metabolism in stem spines may reflect not only metabolic adaptation to drought stress but also the importance of coordinated IAA, serotonin, and melatonin regulation in stem spine drought acclimation.

Two aspects of our study require caution when interpreting the results. First, although the DEG profiles reported here were derived from a rigorously controlled RNA-seq pipeline with sufficient biological replication (n=6), the absence of qPCR validation for the identified DEGs is a limitation of this study. Future studies will aim to validate these key findings using independent sample sets and qPCR assays. In addition, soil physicochemical and microclimatic variables across the three sites were not quantified, which may have confounded the observed responses. Future controlled experiments using standardized soils are needed to disentangle these effects.

## 5 Conclusions

The results indicated that SOD activity in *N. tangutorum* stem spines was significantly greater under Mo than under Mi, suggesting that effective antioxidant defenses were activated to mitigate oxidative damage caused by stress; at the same time, drought reduced chlorophyll content. Integrated transcriptomic and metabolomic analyses revealed species-specific divergence in drought-adaptation strategies at the pathway level: in the isoflavonoid biosynthesis pathway, *A. sparsifolia* mainly improved drought tolerance by modulating coumestrol and glyceollin II accumulation, whereas *N. tangutorum* stem spines exhibited a stage-dependent drought-tolerance mechanism, prioritizing precursor supply to boost genistein production under Mo and shifting to reinforcement of the isoflavonoid pathway itself under S. At the same time, in the tryptophan metabolism pathway, drought adaptation in *N. tangutorum* stem spines mainly relied on regulation of IAA biosynthesis and melatonin-related pathways, thereby jointly achieving antioxidant homeostasis and stress-signal modulation. Revealing the metabolic mechanisms underlying stem spine drought tolerance will not only advance theories of desert plant adaptation, but also provide a foundation for predicting stability changes in desert ecosystems under extreme drought and for formulating conservation and restoration strategies.

## Data Availability

The transcriptomic datasets generated in this study have been deposited in the NCBI Sequence Read Archive (SRA) under BioProject accession number PRJNA1512605. The metabolomic datasets have been deposited in the MetaboLights repository under accession number MTBLS15378 and can be accessed at https://www.ebi.ac.uk/metabolights/MTBLS15378.
