# Supplementary material for: Integrative transcriptomic and metabolomic analyses of the drought response mechanisms of stem spines in desert plants from the Ebinur Lake
Source: Front Plant Sci. 2026 Aug 24;17:1911473. doi: 10.3389/fpls.2026.1911473 (PMC13547513; doi:10.3389/fpls.2026.1911473)
Supplement: Supplementary file 1 [file SupplementaryFile1.docx]

**Supplementary material**

**Integrative transcriptomic and metabolomic analyses of the drought-response mechanisms of stem spines in desert plants from the ebinur lake wetland**

Meiyu Wang ^a,b,^ Wenjing Li ^a,b*^, Hengfang Wang ^a, b^ , Guanghui Lv ^a, b^, Jianhao Li ^c*^

^a^ College of Ecology and Environment, Xinjiang University, Urumqi Xinjiang, 830046, China

^b^ Key Laboratory of Oasis Ecology of Education Ministry, Xinjiang University, Urumqi 830046, China

^c^ College of Geography and Remote Sensing Sciences, Xinjiang University, Urumqi Xinjiang, 830046, China

*Corresponding author: Wenjing Li

E-mail: liwenjing0624@xju.edu.cn

Address: College of Ecology and Environment, Xinjiang University, Urumqi 830017, China

*Corresponding author: Jianhao Li

E-mail: ljh@xju.edu.cn

Address: College of Ecology and Environment, Xinjiang University, Urumqi 830017, China

Fig. S1. Relative water content of stem spines of *A. sparsifolia* and *N. tangutorum.* Uppercase letters (A, B, C) denote the significant differences among drought gradients for *A. sparsifolia*, while lowercase letters (a, b, c) denote those for *N. tangutorum*.

Fig. S2. Pearson correlation coefficient analysis of RNA-seq data

Fig. S3. Overlapping relationship of differential genes in *A.sparsifolia* and *N.tangutorum* stems.

Fig. S4. GO enrichment in the stem spines of *A.sparsifolia*.

Fig. S5 GO enrichment in the stem spines of *N.tangutorum.*

Fig. S6. KEGG enrichment analysis of DEGs in the stem spines of *A.sparsifolia*.

Fig. S7. PCA analysis of metabolomics, A, *A. sparsifolia*; B, *N. tangutorum*.

Fig. S8. Identification of DAM in stem spines of A.sparsifolia. A: OPLS-DA score plot of MoL vs. MiL and SL vs. MiL. B: Venn diagram of DAM

Fig. S9. The number of DAM of *A.parsifolia* and N.*tangutorum*, red : up-regulated; green : down-regulated.

Fig. S10. Hierarchical clustering heat map for correlation analysis of differential genes and differential metabolites in stems of *A.sparsifolia* and *N.tangutorum*.

Fig. S11. KEGG enrichment analysis of the top 20 significant enrichment pathways of DEG and DAM in stem spines of *N.tangutorum*.
